# Improved performance of sequence search approaches in remote homology detection

**DOI:** 10.12688/f1000research.2-93.v2

**Published:** 2014-07-16

**Authors:** Adwait Govind Joshi, Upadhyayula Surya Raghavender, Ramanathan Sowdhamini

**Affiliations:** 1 National Centre for Biological Sciences (Tata Institute of Fundamental Research), Gandhi Krishi Vignyan Kendra Campus, Bangalore, 560065, India; 2 Manipal University, Manipal, Karnataka, 576104, India

## Abstract

The protein sequence space is vast and diverse, spanning across different families. Biologically meaningful relationships exist between proteins at superfamily level. However, it is highly challenging to establish convincing relationships at the superfamily level by means of simple sequence searches. It is necessary to design a rigorous sequence search strategy to establish remote homology relationships and achieve high coverage. We have used iterative profile-based methods, along with constraints of sequence motifs, to specify search directions. We address the importance of multiple start points (queries) to achieve high coverage at protein superfamily level. We have devised strategies to employ a structural regime to search sequence space with good specificity and sensitivity. We employ two well-known sequence search methods, PSI-BLAST and PHI-BLAST, with multiple queries and multiple patterns to enhance homologue identification at the structural superfamily level. The study suggests that multiple queries improve sensitivity, while a pattern-constrained iterative sequence search becomes stringent at the initial stages, thereby driving the search in a specific direction and also achieves high coverage. This data mining approach has been applied to the entire structural superfamily database.

## Introduction

Protein sequence databases have grown enormously in recent times. Understanding protein homology within such huge sets of sequences requires tracing the divergence by mutation, substitution, insertion and deletion of residues ^1, 2^. Homologous proteins reflect similarity at sequence and structural levels, implying functional similarity ^3^. This level of similarity broadens into the superfamily and the ways to deduce such relationships differ for both protein sequence and structure information ^4, 5^. There are different databases that organize sets of homologous proteins or protein superfamilies based on protein sequence and structure. These databases primarily employ protein domain information present in a sequence or structure. SCOP is a database that organizes the protein structural domain data in different hierarchical levels based on structural and functional information ^6^. Many sequence search strategies use SCOP domains as a starting point for homology detection, focusing mainly at the superfamily level ^7, 8^. Structure-based classification is helpful to explore sequence space and helps in functional assignments by association of protein sequences ^9^.

Of the several methods developed for protein homology detection, the popular BLAST ^10^ algorithm uses heuristics to search sequences and is able to detect close homologues, but fails in a few instances to establish relationships between distantly related proteins. To detect remote homologues, several methods such as, PSI-BLAST ^10^ based on profiles, Hidden Markov Model (HMM)-based methods like HMMSEARCH and Jackhmmer ^11, 12^, pattern-based methods like PHI-BLAST ^13^, intermediate sequence search methods such as Cascade PSI-BLAST ^14^ and phylogenetic tree based searches like Treesearch ^15^ have been developed. Methods such as CHASE incorporate some of the above methods in a combined manner to reinforce the sequence search ^16^. Each of the above methods can be optimized for better performance by customizing their parameters and the way they are implemented for sequence searches. For instance, PSI-BLAST is an iterative PSSM (Position Specific Scoring Matrix)-based remote homology detection method. In Cascade PSI-BLAST the search is iterated for several generations in a cascaded manner to improve remote homology detection ^14^. It is important to select appropriate start points for sequence searches, especially for protein families and superfamilies, as different start points can result in different coverage. Park and co-workers have shown that remote homology detection is enhanced threefold for a set of related sequences in the form of a profile than merely searching with a single sequence as a query ^17^. Anand and co-workers emphasized the use of multiple PSSMs as better detectors of remote homologues compared to a single query ^18^. Thus, a search strategy can be designed to improve remote homology detection by choosing appropriate method(s) and starting point(s) for the search and by further optimizing the parameters.

We have considered multi-member superfamilies from the PASS2 database ^19^. PASS2 is a database of structural alignments of protein domains in a SCOP superfamily which share less than 40% mutual sequence identity. The strategy lies in using all the members of PASS2 superfamily, i.e., multiple members, and searching against the NR-Db (Non Redundant Database) available at the NCBI protein resource. We present our analysis based on a multiple query approach (MQ) for PSI-BLAST and PHI-BLAST. While using PHI-BLAST, for each query we obtain a set of patterns to initiate multiple searches per query, thereby adding an extra dimension for multiple patterns. The search approaches are evaluated at different coverage levels and a comparison of the two approaches using the two methods is presented. The methods are then applied to the entire PASS2 database.

## Materials and methodology

### Dataset

Different structural classes of proteins described in SCOP (version 1.75) were considered for remote homology detection. PASS2 ^19^ based on the SCOP database and the ASTRAL compendium ^20^, uses protein structural entries from the SCOP superfamily with less than 40% mutual sequence identity. Three superfamilies each from four structural classes from the PASS2 database (2008 version) were selected for this study ( Table 1). Each superfamily contains a varied number of members, with some containing a single family and others with more than one family. The classification of families is based on SCOP hierarchies; PASS2 does not consider this classification. Therefore, different members within a PASS2 superfamily may be listed in different families in SCOP. The sequence search strategy devised, described below, was implemented for data mining the sequence homologues for structural superfamilies. Structural members from all the 1961 PASS2 superfamilies were used to search for sequence homologues.

**Table 1.** List of 12 PASS2 superfamilies considered for sequence searches.

Class	SCOP Code	No. of members	No. of SCOP families	Size	Name of superfamily as in SCOP database
ALL α	47336	7	3	86	Acyl carrier protein-like
	47565	5	1	120	Insect pheromone/odorant- binding proteins
	48345	3	3	230	A virus capsid protein alpha helical domain
ALL β	51101	3	1	147	Mannose-binding lectins
	50203	10	2	100	Bacterial enterotoxins
	51069	4	1	248	Carbonic anhydrase
α AND β	55031	7	1	109	Bacterial exopeptidase dimerisation
	55307	5	1	146	Tubulin C-terminal domain-like
	55239	4	1	119	RuBisCo-small subunit
α OR β	51971	9	3	243	Nucleotide-binding domain
	51679	6	4	343	Bacterial-luciferase-like
	51351	3	1	243	Triose phosphate isomerase

### Homologue detection

**Figure 1.**
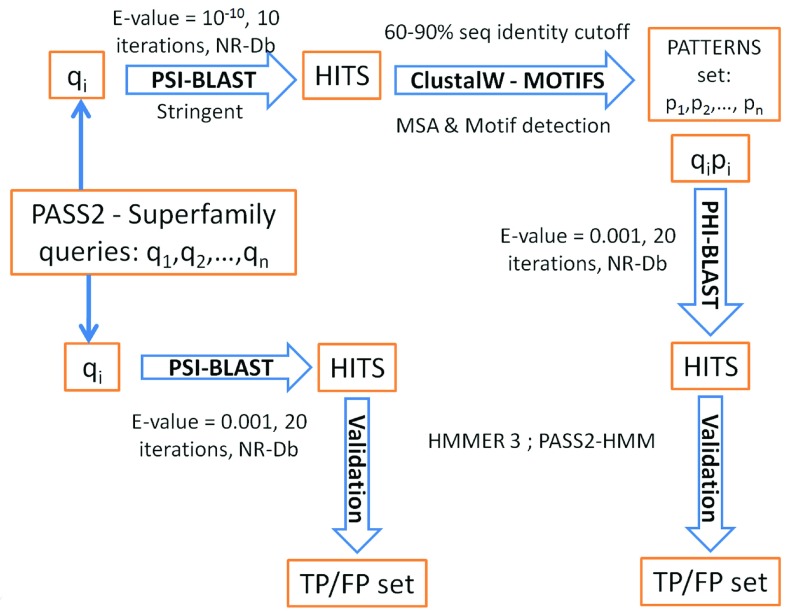
Flowchart of workflow for the sequence search strategy.

Each member of the PASS2 superfamily was selected as a query for homologue detection and used to search against the NR-Db available at the NCBI ftp site ( ftp://ftp.ncbi.nih.gov/blast/db/FASTA/nr.gz). Two popular methods, PSI-BLAST and PHI-BLAST, from the BLAST 2.2.23+ package, were used for sequence searches ( ftp://ftp.ncbi.nih.gov/blast/executables/blast+/2.2.23/ncbi-blast-2.2.23+-x64-linux.tar.gz) (see Figure 1 for flow-chart).

PSI-BLAST was used for each query until convergence or a maximum of 20 iterations. The expectation value threshold (E-value: parameter –e) and inclusion threshold (parameter –h) were both set to 0.001. In all the iterations, all the alignment regions from each of the hit sequences were considered for further validation steps. Following this, a set of all unique sequence homologues (hits with a unique GenInfo Identifier (GI)) was recorded for each member. Sets of hits from all the members in a superfamily were pooled and filtered to generate a cumulative set of unique GIs for each superfamily. Employing all members for homology detection for a superfamily is essentially the multiple query (MQ) approach in the sequence search strategy. For all the PASS2 superfamilies, data mining was carried out using this MQ approach and PSI-BLAST on the latest NR-Db version (June, 2012 release) to populate a sequence homologue database for structural superfamilies ^7^. PHI-BLAST requires a query and a pattern pair as an input. A set of patterns was identified for each query and each query-pattern pair was used for the PHI-BLAST search. The parameters and their values were maintained as in PSI-BLAST searches. The use of multiple patterns for all the multiple queries (MPMQ approach), for superfamily level homology detection was tested on the 12 PASS2 superfamilies.

### Pattern generation

Homologues were searched for query sequence patterns, using stringent PSI-BLAST criteria (maximum 10 iterations, 10 ^-10^ E-value), in NR-Db. A set of hits with 60–90% sequence identity was selected for the pattern generation process. For queries with <10 hits, the sequence identity window was relaxed to 40–95% so that there were enough sequence homologues to deduce a conserved pattern. These hits were aligned using ClustalW 2.0 ^21^. An in-house program, MOTIFS (the program can be made available upon request), was used to identify motifs from the Multiple Sequence Alignment (MSA). The program accepts a MSA of protein sequences as input and employs a DAYHOFF-type amino acid exchange matrix to score amino acid replacements for every pair of sequences. The average pairwise amino acid exchange scores are recorded at every alignment position. In general, an alignment position with a high score means a highly conserved position. A motif is reported for a stretch of three or more consecutive positions with complete conservation or favorable substitution (based on an identical or similar residue in a given position in the MSA and the average score). For all such identified motifs, the stretch of amino acids from all the sequences is reported with their length and position corresponding to the first sequence in the alignment. The motif generated is screened for the following conditions:

- Length of the motif should be ≥ 3
- At each position, there should be only ≤ 3 different amino acid preferences (reported by MOTIFS); if not, then that position is denoted as ‘X’, where ‘X’ refers to any amino acid at that position
- When there is an occurrence of two consecutive ‘X’s (variable residues), the pattern is divided into two separate patterns
- No pattern starts or ends with ‘X’
- Any pattern can have a maximum length of 15 amino acid positions, since above this length there is little increment in the number of hits obtained. If length of a pattern is >15 amino acid positions, it is broken into subsets of patterns which meet all the above conditions and has a maximum length of 15.

After the motif passes through all the above filters, it is converted into a PROSITE format pattern, and used for PHI-BLAST along with its query ^22^.

### Validation

The PASS2 database contains structure-based sequence alignments which are used to build a HMM for each superfamily. Since these HMMs are built using structural alignments, they are highly sensitive in validating the sequence homologue obtained for a superfamily member as a true positive (TP). A library of all PASS2 superfamily HMMs was consulted using HMMSCAN, from the HMMER 3.0 package ( ftp://selab.janelia.org/pub/software/hmmer3/3.0/hmmer-3.0-linux-intel-x86_64.tar.gz), to validate all the sequence homologues through either of the sequence approaches (MQ and MPMQ) discussed above ^11^. This approach of validation had earlier been effective in 80% of the examples ^23^.

In the process of data mining for all PASS2 superfamilies using the MQ approach, additional validation was used for the homologues which failed to associate with the correct superfamily HMM. A single query HMM was built for all PASS2 members which were grouped together to generate a library. Thus, an additional validation using single-query HMM was carried for all PASS2 superfamilies. All the HMMSCAN runs were performed with an E-value threshold of 0.01.

### Coverage of sequence search approaches

Coverage for both of the sequence search approaches was based on different levels. Firstly, a cumulative set of validated hits (Cum-TP) obtained for each superfamily should contain all the PASS2 members comprising that particular superfamily. Secondly, hits corresponding to the sequences of all the SCOP members for a given superfamily should be part of the Cum-TP set. Since, PASS2 contains superfamilies where no two members within a superfamily had >40% sequence identity, the coverage at the SCOP level reflects the sensitivity of the search strategy. Any cross-superfamily hits detected were considered as false positives (FP), even if they belong to the same fold. Thirdly, since sequences of all structural entries present in Protein Data Bank (PDB) obtained as hits are not classified into SCOP, all structural entries were inspected through HMM validation whether they associate to a single PASS2 superfamily HMM with no cross-superfamily associations. Finally, all the protein sequence homologues devoid of the above mentioned levels, with no structural information, were validated by HMMSCAN.

In each superfamily, a set of cumulative true positives (Cum-TP) was formed with hits having unique GI obtained from different members. Therefore, from the set of all hits obtained for a superfamily (All Hits), the number of validated hits, i.e. Cum-TP, was used to calculate positive prediction value (PPV) for all 12 superfamilies in both approaches. The PPV is calculated as follows:

$$PPV=\left(\frac{Cum_{TP}}{All\;Hits}\right)*100$$

A member with highest number of TPs was identified as a best representing sequence (BRS) and its TP count was also recorded (BRS-TP) for all superfamilies. Corresponding to Cum-TP, a ratio of BRS-TP/All Hits was also calculated. Using this value and Cum-TP, a percentage gain in the coverage (PGC) was recorded:

$$PGC=\left(\frac{Cum_{TP}–BRS_{TP}}{Cum_{TP}}\right)*100$$

The overlap between coverage achieved by the four cases – MPMQ approach, MQ approach, BRS from PHI-BLAST and PSI-BLAST was assessed by plotting a Venn diagram ^24^. Following these fourcases, all the validated hits obtained for all the 12 superfamilies were segregated into 4 sets and used for plotting the Venn diagram.

## Results

The sequence search strategy devised for remote homology detection was tested and implemented.

### MQ approach for sequence search

***Selection of parameters.*** 12 structural superfamilies were considered for testing the sequence search strategy. These superfamilies span different classes (α, β, α/β, α+β) of proteins, as per the SCOP definitions. In the MQ approach, all the members from each of the 12 selected superfamilies were used as inputs for PSI-BLAST to search against the NR-Db. The performance of PSI-BLAST was optimized for different parameters. The E-value (parameter –e) and inclusion threshold (parameter –h) were optimized to 0.001, after testing it on a range of 1 to 10 ^-10^. An optimized E-value ensures better coverage with fewer FP. The number of iterations (parameter –j) was set to 20, after testing for values 5, 10 and 20. Some queries may converge within 5 iterations where the superfamily was less diverse as in the case of 50203 (Bacterial enterotoxin). However, for certain superfamilies like 55239 (RuBisCo-small subunit), none of the members could converge searches within 20 iterations explaining the abundance of sequence homologues for such proteins in the NR-Db. The rest of the parameters were set to default.

**Figure 2.**
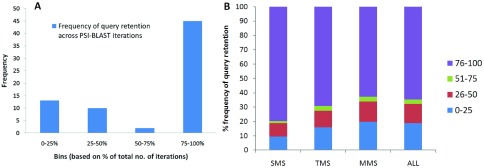
Frequency of retention of query until 25%, 50%, 75% or 100% of iterations of PSI-BLAST is represented as four bins (0–25%, 26–50%, 51–75%, 76–100% respectively). ( **A**) Number of member queries observed per bin for 12 superfamilies and ( **B**) A stacked bar plot for all percentage query retention bins observed for all superfamily members in PASS2 database and also for the different sets of SMS (Single-Member Superfamily), TMS (Two-Member Superfamily) and MMS (Multi-Member Superfamily).

***Query retention.*** In the profile-based iterative (PSI-BLAST) run, the sensitivity and specificity depends upon the quality of the PSSM generated per iteration. If the PSSM gets corrupted, then the PSI-BLAST search drifts to FPs by inclusion of non-homologous sequences. This can be traced by retention of the query in the PSSM throughout the iterations of a PSI-BLAST run. A query retained until the end reflects good optimization of search parameters and little corruption of the PSSM. To study the query retention for each member in a superfamily, the presence of query was inspected in all iterations for the PSI-BLAST run. The number of iterations was divided into four bins as 25%, 50%, 75% and 100% of total iterations for which the PSI-BLAST run lasted or converged. If a PSI-BLAST run converged at the 16 ^th^ iteration, upper limits for these four bins were 4 ^th^, 8 ^th^, 12 ^th^ and 16 ^th^ iterations, respectively. The query was inspected for the occurrence in any of the four bins for all members of a superfamily. The frequency of queries last observed in any of the four bins is plotted in Figure 2-A. It reflects that out of a total of 70 queries from all 12 superfamilies, 45 queries were retained until the last iteration and 23 queries were not retained beyond 50% of iterations. However, it was observed that close homologues of the member query (sequence identity >90%) were retained until the last bin, thereby driving the PSI-BLAST search in the correct direction. Query drift was observed in only two out of the total 70 members from 12 different superfamilies.

In the data mining of all structural superfamilies within the PASS2 database using the MQ approach, a similar trend was observed for the entire set of 10569 queries arising from 1961 superfamilies. 65% of queries were observed in the fourth bin, thereby ensuring the parameters selected for this scale-up of a MQ approach were acceptable. At times, the loss of query in the PSSM could still be beneficial, if the resultant PSSM is enriched by neighboring families, thus improving the coverage at the superfamily level. The PASS2 database includes the classification of superfamilies as single-member (SMS), two-member (TMS) and multi-member (MMS) based on the number of members in each superfamily. A distribution for the query retention in the four bins for these sections is shown in Figure 2-B. 68% of MMS queries have >50% query retention. However, in the MQ approach, few of the members in MMS are retained until the fourth bin to ensure better sequence search at the superfamily level.

### MPMQ approach for sequence search

Each member and pattern pair was used for sequence searches using PHI-BLAST. Similar to PSI-BLAST, the E-value (parameter -e) and inclusion threshold (parameter -h) were optimized to 0.001. The maximum number of iterations allowed was set to 20. While generating the patterns, it was ensured that patterns are specific and stringent to query. The length of the patterns generated varies from 3 to 40 amino acid positions; however during sequence search using PHI-BLAST, the pattern length of maximum 15 amino acid positions was used. Longer lengths reflect the long conserved stretches in the homologues selected for pattern generation. Multiple patterns for each query and multiple queries per superfamily (MPMQ approach) were used in the sequence searches. This gives rise to multiple start points to search in sequence space for each superfamily. Although PHI-BLAST essentially follows an iterative protocol like PSI-BLAST, the hits identified in the first iteration differ as they are constrained to have the pattern specified along with the query. This ensures that the hits will be used to create the first PSSM for the rest of the iterative search process.

### Validation and coverage

The sequence hits obtained through MQ and MPMQ approaches were validated for TP and then inspected for the coverage at different levels for all 12 superfamilies.

***Coverage for PASS2 members.*** Every member was examined for its coverage of all the PASS2 members in the sequence search. All the members achieved full coverage when searched in the cumulative set of hits derived from sequence searches of all the PASS2 members. Every member of the two superfamilies, RuBisCo small subunit (SCOP code: 55239) and mannose-binding lectins (SCOP code: 51101), could identify all other members. But for the rest of the superfamilies, every member was unable to identify all the PASS2 members. Although, single members in such instances were unable to cover all the PASS2 members cumulatively, presence of all members gave rise to 100% coverage, thereby stating the importance of the use of multiple queries (MQ).

**Figure 3.**
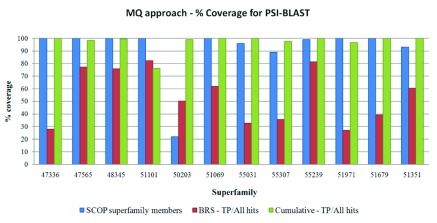
Coverage of 12 superfamilies for the PSI-BLAST sequence search. The blue bars correspond to the percentage of SCOP members identified as hits. The red bars correspond to percentage ratio of TP (True Positives) identified by BRS (Best Representative Sequence) and All Hits (BRS-TP/All Hits). The green bars correspond to percentage ratio of Cumulative TP identified for a superfamily and All Hits (Cum-TP/All Hits).

***Coverage for SCOP superfamily members.*** The coverage for all the SCOP superfamily members was inspected. The ratio of the number of sequence homologues of SCOP superfamily entries identified as TP to the total number of SCOP superfamily members is plotted as shown in Figure 3 (blue bars). Almost all of the superfamilies obtained >90% coverage for all SCOP superfamily members. The only exception was the Bacterial enterotoxins superfamily with two families (SCOP code: 50203), where coverage was 25%. In the PASS2 database, this superfamily contained nine members from one SCOP family, and could identify most of its SCOP family members. But none of the members could identify cross-family members. However, there was only one member from the other family which was not sufficient to identify all the family members. This lack of coverage can be overcome if more members are included from the second family. For such a diverse superfamily, it is apparent that unless we use an MQ approach, it is difficult to achieve a reasonable coverage. In the rest of the superfamilies, some members were able to identify cross-family members while some could not identify any cross-family member. In either case, all of the SCOP family members were covered. Cumulatively, all PASS2 members are strong enough to cover most of the SCOP superfamily members underlining the high sensitivity of MQ approach.

***Coverage for the structural (PDB) entries.*** The sequences of structural entries present in PDB were part of the All Hits set. These are newer entries which are yet to be accounted in the SCOP database. After PASS2-HMM validation, they were inspected for which superfamily they belong to and if they could associate to the query member’s superfamily. There were no cross-superfamily connections, highlighting the specificity of the sequence search through the MQ approach.

***Coverage with respect to the TP sequence homologues.*** While following the MQ approach over a huge database like NR-Db, it is necessary to achieve a maximum number of homologues (TP) and minimum unrelated sequences (FP). The use of multiple queries ensures a high TP count, while appropriate stringency of the E-value restricts occurrence of FPs. Following validation based on PASS2 HMMs, a set of Cum-TP was obtained and a ratio of Cum-TP/All Hits was calculated. Similarly, a BRS-TP/All Hits ratio was also calculated. Both these values are expressed as percentages as shown in Figure 3 (green and red bars respectively). The percentage of SCOP entries identified is also represented in Figure 3 for each superfamily. Considering the Cum-TP/All Hits ratio, Figure 3 shows the extent of coverage achieved by the BRS. It is clear that none of the BRS could achieve more than an 80% TP share of all the TPs identified at superfamily level. The average coverage for BRS across all superfamilies was ~50%. However, the Cum-TP/All Hits ratio was >90% for most of the superfamilies. In most of the cases, the false positives were hits which could not get associated with any PASS2-HMM, but did not associate with any other superfamily apart from query superfamily. This may partly be due to the fact that the superfamily was so diverse that the PASS2-HMM itself was getting noisy due to the diversity or else the hit obtained was too distant to get validated using the HMM validation cut-offs. On average, false positives were <3%. The results strongly suggest that the MQ approach to sequence data mining helps to increase coverage at the complete superfamily level. For superfamilies which have multiple families (47336, 55031), none of the BRS could obtain a BRS-TP/All Hits ratio >35%, but cumulative coverage by all queries for a complete superfamily is excellent (100% Cum-TP/All Hits ratio). Even in the case of a single-family superfamily (55031 - Bacterial exopeptidase dimerisation domain superfamily), all the members have attained a 100% Cum-TP/All Hits ratio.

### MPMQ approach outperforms MQ approach

The coverage of the MPMQ approach for all 12 superfamilies was equal to or better than the MQ approach at each level discussed above. All the PASS2 members were identified cumulatively by all the members. The coverage increased for all the superfamilies with respect to the SCOP superfamily (except for the 50203 superfamily which performed poorly even in the MQ approach). The sequences of structural (PDB) entries identified were associated with the query superfamily PASS2-HMM. Similar to PSI-BLAST, PHI-BLAST results also revealed that the use of multiple queries are beneficial for achieving better coverage at PASS2, SCOP and PDB level.

The MPMQ approach was contrasted with the MQ approach ( Table 2). The high PPV value suggests that both approaches were able to discern between the TP and FP. The MPMQ approach has relatively more start points than the MQ approach, employing pattern-constrained PHI-BLAST. The BRS identified in PSI-BLAST was also the same in the PHI-BLAST results for most of the superfamilies. However, the performance of BRS from PHI-BLAST using multiple patterns (MP) was even better for some of the superfamilies than MQ approach wherein all the queries were used through PSI-BLAST for sequence search. There was an increase in the number of TP identified for BRS PHI-BLAST as against MQ in 5 out of 12 superfamilies. The TP count for all 12 superfamilies was highest for MPMQ with better PPV for 10 out of 12 superfamilies than MQ approach.

**Table 2.** Comparison of MQ (Multiple Query) and MPMQ (Multiple Patterns – Multiple Query) approaches for 12 superfamilies (SF – Superfamily, TP – True Positive, Cum-TP – Cumulative true positives, PPV – Positive Prediction Value, bold faced entries are BRS (Best Representative Sequence).

SF	Members	MQ				MPMQ			
		*Hits*	*TP*	*Cum-TP*	*PPV*	*Hits*	*TP*	*Cum-TP*	*PPV*
48345	d1bvp11	99	97	468	0.99	101	98	502	0.99
	**d1qhda1**	**356**	**356**			**390**	**390**		
	d1uf2c1	15	15			16	16		
47565	d1c3ya	722	706	1008	0.98	1126	1089	1259	0.95
	d1ooha	702	701			1089	1056		
	**d1p28a**	**807**	**792**			**1162**	**1151**		
	d1r5ra	731	723			1155	1119		
	d2p70a1	681	678			1109	1105		
47336	d1dnya	857	851	3368	0.99	1328	1328	4595	0.99
	**d1dv5a**	**945**	**944**			**1494**	**1491**		
	d1klpa	814	814			1197	1197		
	d1nq4a	750	741			1312	1310		
	d1t8ka	672	672			1153	1153		
	d1vkua	637	637			10	10		
	d2pnga1	905	894			1399	1396		
51069	d1jd0a	581	581	1032	1	1154	1154	1508	1
	d1kopa	627	627			1346	1346		
	d1luga	572	572			1336	1336		
	**d2znca**	**638**	**638**			**1360**	**1360**		
50203	d1an8a1	94	94	433	0.99	95	95	436	0.99
	d1enfa1	220	218			221	221		
	**d1esfa1**	**220**	**219**			**221**	**221**		
	d1et9a1	5	5			6	6		
	d1eu3a1	94	94			58	58		
	d1m4va1	101	101			102	102		
	d1prtb1	16	14			17	15		
	d1ty0a1	94	94			95	95		
	d1v1oa1	101	101			102	102		
	d3seba1	185	184			186	186		
51101	d1c3ma	599	496	471	0.76	607	501	506	0.81
	d1ouwa	601	499			597	487		
	**d1ugx1**	**602**	**507**			**616**	**507**		
55307	d1rq2a2	621	585	1977	0.97	1254	1184	3545	0.98
	d1tuba2	548	548			1081	1081		
	d1tubb2	598	598			1093	1093		
	**d1w5ba2**	**734**	**723**			**1291**	**1279**		
	d1w5fa2	692	687			1255	1250		
55031	**d1cg2a2**	**1186**	**1186**	3629	0.99	**2749**	**2749**	5615	0.99
	d1fnoa3	531	530			675	671		
	d1r3na2	615	615			1284	1283		
	d1vgya2	657	657			1758	1758		
	d1ysja2	807	807			1193	1193		
	d1z2la2	642	642			1156	1155		
	d2q43a2	941	941			1501	1501		
55239	**d1bwvs-**	**691**	**691**	849	1	1084	1084	1161	1
	d1rlbi-	580	580			1093	1093		
	d1svdm1-	592	592			1115	1115		
	d1wdds-	596	596			**1124**	**1124**		
51351	**d1aw1a**	**819**	**819**	1356	1	1723	1723	2183	0.99
	d1hg3a	780	780			**1818**	**1815**		
	d1n55a	727	727			1578	1578		
51679	d1ezwa-	1180	1180	3582	1	**2440**	**2440**	5239	1
	d1luca-	1312	1312			2025	2025		
	**d1lucb-**	**1401**	**1401**			2225	2225		
	d1nqka-	814	814			1803	1803		
	d1rhca-	1014	1014			2269	2269		
	d1tvla	661	661			1172	1172		
51971	d1c0pa1	1046	929	4215	0.9	1813	1505	8031	0.9
	d1cjca2	966	964			1941	1941		
	d1h7wa4	864	864			1504	1503		
	d1i8ta1	631	619			**2256**	**2071**		
	d1lqta2	1011	1011			2144	2143		
	**d1o94a3**	**1182**	**1181**			2044	2044		
	d1ps9a3	577	575			1248	1248		
	d1usja1	626	615			1772	1674		
	d1ve9a1	808	722			2168	1646		

**Figure 4.**
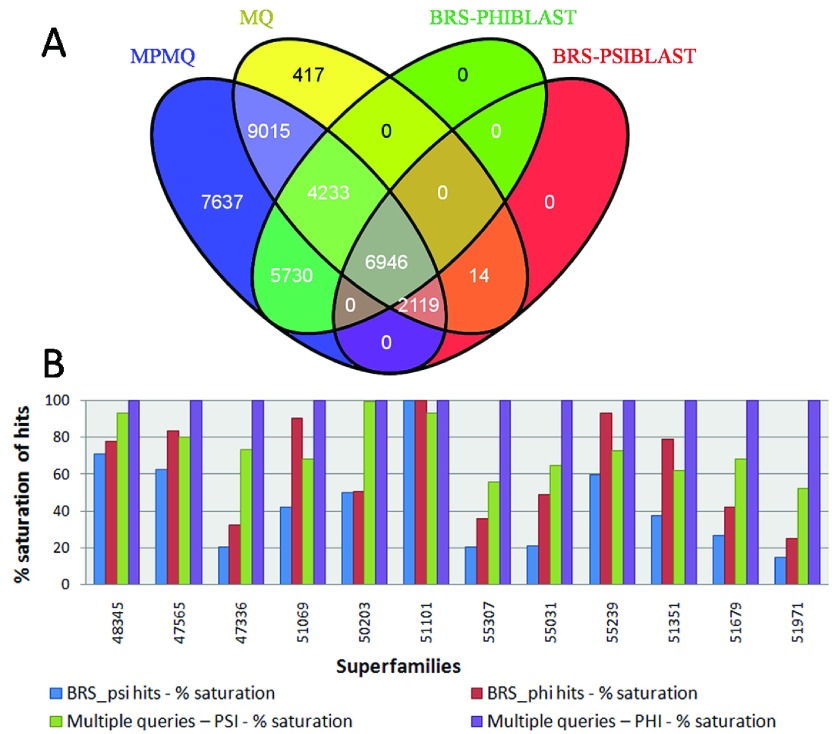
( **A**) Venn diagram representing four sets MPMQ (Multiple Pattern-Multiple Query), MQ (Multiple Query), BRS (Best Representative Sequence) from PHI-BLAST and BRS from PSI-BLAST comprising of all hits combined for 12 superfamilies. ( **B**) Comparison of MPMQ and MQ with respective BRS-TP (Best Representative-True Positives). The blue and green bars correspond to TP obtained for BRS (BRS-TP) and cumulatively (Cum-TP) using all queries in the superfamily respectively using PSI-BLAST. The red and purple bars correspond similarly to TP obtained for BRS (BRS-TP) and cumulatively (Cum-TP) for all queries in the superfamily respectively using PHI-BLAST. All values are % saturation considering Cum-TP for PHI-BLAST (highest number of TP) as the saturation value for each superfamily.

Corresponding to the four approaches – MPMQ, MQ, BRS PHI-BLAST and BRS PSI-BLAST, the total hit count with MPMQ was highest for each superfamily. Hence, all the hits obtained were combined for all 12 superfamilies together. Following the above mentioned four approaches, the total hits-set (36111 hits), was segregated into four sets (MPMQ = 35680, MQ = 22744, BRS PHI-BLAST = 16909, BRS PSI-BLAST = 9079) and plotted as a Venn diagram ( Figure 4-A). The total hit sets obtained by BRS of both PSI-BLAST and PHI-BLAST were subsets of the MQ and MPMQ approaches, respectively. However, it was interesting to find that 98% of the hits identified through MQ approach were covered by MPMQ, emphasizing the performance of MPMQ over MQ approach. Moreover, MPMQ approach covered 37% more hits which were not identified by MQ approach. The BRS PHI-BLAST approach alone was able to identify 15% more hits than MQ approach, suggesting that employment of BRS PHI-BLAST approach alone can augment the MQ approach for a gain in coverage.

The MPMQ approach covers 99% of the total hit set. This was chosen as 100% coverage (% saturation value) to compare performance of rest of the 3 cases (viz. MQ approach, BRS PHI-BLAST and BRS PSI-BLAST) for each superfamily, separately. The performance was compared by plotting percentage coverage achieved using the BRS-TP/All Hits and Cum-TP/All Hits ( Figure 4-B). The Cum-TP/All Hits was highest in all superfamilies for the PHI-BLAST runs. This was chosen as 100% coverage (% saturation), to compare performance of BRS in MQ and MPMQ and Cum-TP/All Hits for the MQ approach. The Cum-TP/All Hits (MPMQ) was almost double for 55307 and 51971. This difference between MPMQ and MQ approaches was >20% for 9 out of 12 superfamilies. Although the MPMQ approach has promising results in comparison to the MQ approach, it is computationally very expensive. Each member in a superfamily generates multiple patterns, hence the higher the number of query-pattern pairs means the number of PHI-BLAST runs to be performed for the given superfamily increases proportionally.

The performance of BRS in both approaches was also inspected. BRS for MPMQ achieved close to 80% coverage in 9 out of 12 superfamilies, with an exceptionally low value of 25% for the 51971 superfamily (a multi-family superfamily). Considering multi-family superfamilies (47336, 50203, 51679, 51971), the BRS of MPMQ could not cross 50% coverage compared with the Cum-TP/All Hits of MPMQ. This reinforces and lends support to the strategy of using multiple queries for sequence searches at the superfamily level. The MPMQ BRS was able to identify more TPs than Cum-TP obtained through MQ for the 51069, 55239, 51351 superfamilies, which are single-family superfamilies. This implies that BRS in isolation, through PHI-BLAST, can drive sequence searches attaining better coverage in such superfamilies, achieving a trade-off between coverage and computational time. However, performance of BRS is highly superfamily-specific.

### Implementation of MQ approach for data mining

**Figure 5.**
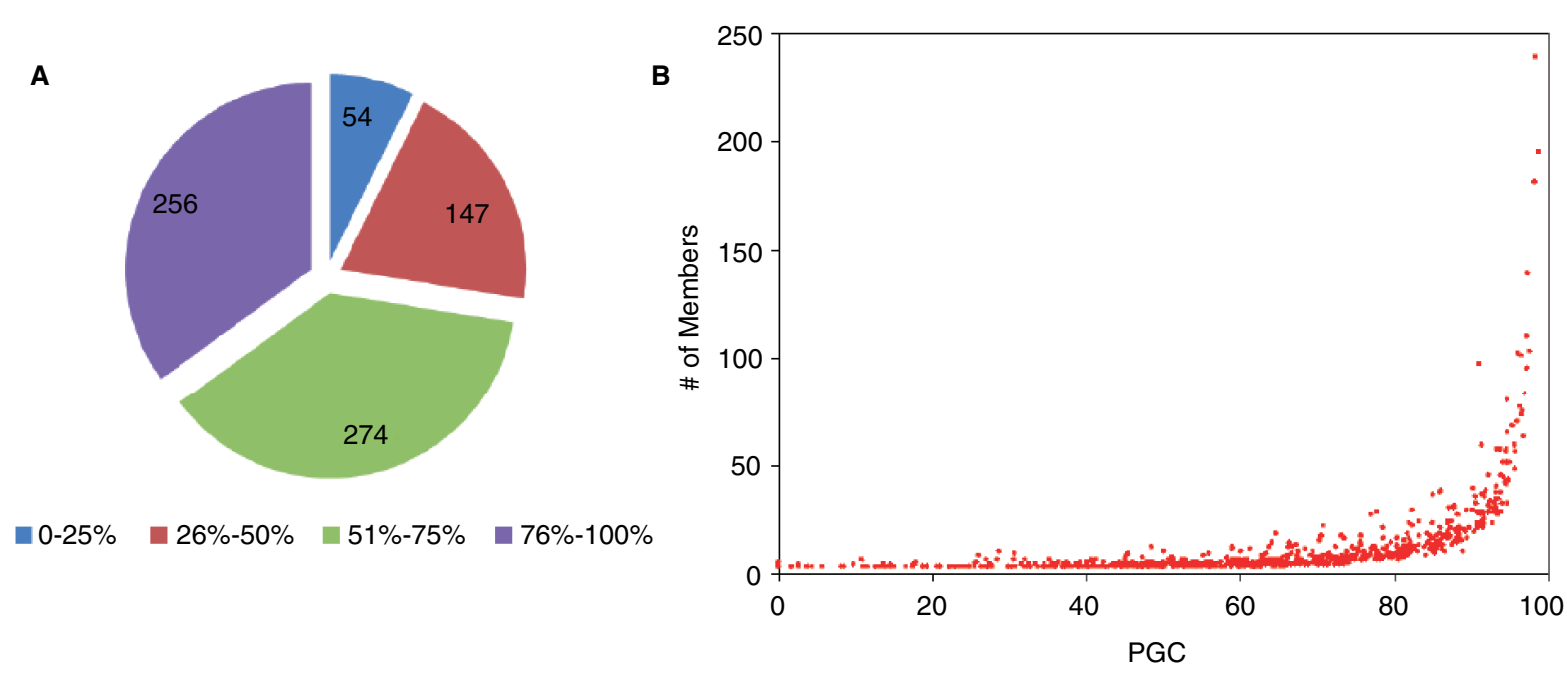
( **A**) Frequency of superfamilies based on PGC (Percentage Gain in Coverage) value divided into 4 bins (0–25%, 26–50%, 51–75%, 76–100%). ( **B**) The PGC value plotted against number of members for all superfamilies.

GenDiS (Genomic Distribution of protein structural domain Superfamilies) is our previous database of sequence homologues based on PASS2, where sequence homologues for structural members are organized with respect to their genomic distribution at the superfamily level ^7^. In an attempt to populate GenDiS, the MQ approach was implemented to search in sequence databases starting with all the 1961 PASS2 superfamilies. PASS2 classification of SMS, TMS and MMS relies on the number of constituent members in a superfamily. For each superfamily a percentage gain in coverage (PGC) value due to improvised strategies, in comparison with simple PSI-BLAST runs, was calculated based on the preliminary results on 731 MMS (see Methods). The PGC was divided into four quarters and the frequency of superfamilies belonging to each quarter is shown in Figure 5-A. It was found that 532 superfamilies obtain >50% PGC. This observation strongly suggests the necessity of the MQ approach for remote homology detection, where the BRS alone collects fewer homologues. The MQ approach has to be implemented, especially for huge databases like NR-Db, to obtain improved coverage at the superfamily level. An inspection of the relation between number of members and PGC for MMS is shown in Figure 5-B, revealing an increase in PGC value with a rise in the number of members. The PGC values are extremely high for diverse and large superfamilies with more than 30 members.

## Discussion

We have employed structural entries from the PASS2 database to understand and improve sequence data mining techniques, where the searches are specifically meant for detection of distant homologues ^19^. SCOP is a good benchmark dataset to evaluate many homology detection methods ^6^, but removing redundancy with respect to similar entries reduces the search time. However, with the use of the MQ approach for searching sequence space there is substantial improvement in coverage, albeit with an increase in search time. Use of PASS2 superfamily members as query is a trade-off, between use of all the SCOP members as query (ideal for best coverage) and a single query for the entire structural superfamily (ideal for saving computational time).

MQ grants an additional advantage of coverage, which is revealed by considering coverage at different levels. A multi-member SCOP superfamily like 48345 (a virus capsid protein alpha helical domain), with multiple families (Orbivirus capsid [SCOP code: 48346], Phytoreovirus capsid [101395] and vp6, the major capsid protein of group A rotavirus [63596]), where each query gives an exclusive set of hits for each query (representing its SCOP family) through both the methods, use of multiple queries is inevitable to achieve a better or even complete coverage. Similarly, another multi-member, multi-family superfamily 51971 (Nucleotide-binding domain) contains nine PASS2 members divided among three SCOP families (N-terminal domain of adrenodoxin reductase-like [SCOP code: 51972] - five members, D-aminoacid oxidase, N-terminal domain [51979] - two members and UDP-galactopyranose mutase, N-terminal domain [69427] - two members). In this superfamily, PASS2 members of one family could not identify PASS2 members from other family. Owing to the diverse nature of this superfamily, it is necessary to adopt the MQ approach for achieving better coverage. Further, for this superfamily, MPMQ approach provided multiple start points and a two-fold increase in coverage as compared to MQ approach. However, it was not possible for PASS2 members to establish a cross-family connection. Therefore, choice of multiple queries with inclusion of their respective multiple patterns increases the overall coverage for diverse superfamilies. These findings comply with previous findings based on different search methods ^17, 25^.

To date, many methods and approaches have been standardized for sequence searches at different levels of database complexity ^8, 16– 18, 25^. PSI-BLAST is in many instances a primary choice for homology detection. Efforts by different groups have enhanced the usage of PSI-BLAST, improving its sensitivity in remote homology detection ^14, 26^. PHI-BLAST, employing an initial pattern–hit initiation, enhances the search method before continuing over profile-based searches in subsequent iterations ^25^. In the present study this has been shown to increase the coverage by >20% for many of the selected superfamilies. The simultaneous use of different methods greatly improves coverage, and the combinations of other methods needs to be investigated.

The choice of BRS identified from MQ may provide a better option to run PHI-BLAST for a single query. This is beneficial considering an increase in coverage, but it is necessary to acknowledge that, multiple queries and their multiple patterns increase computational time tremendously for a given superfamily. Although for a multi-member, multi-family superfamily, this may not effectively work, this approach can serve as a good trade-off between coverage and computational time for a superfamily with a single family. Also, for a very large superfamily (>20 members), the BRS-PHI-BLAST will be a good option to choose, however the choice of any of the discussed approaches for sequence search becomes superfamily specific.

## Conclusion

In the post-genomic era, there is a strong need to devise sequence search strategies for effective functional annotation. Functional annotation transfer and convincing establishment of sequence relationships are bottlenecks at low sequence identities. Hence, there is a constant quest for a robust computational program which is greedy on coverage and stringent in eliminating false positives. Remote homology detection through sequence searches has been addressed by various methods and approaches. Considering a protein structural superfamily, when an attempt to view its spread in the sequence space is made, one can resort to various methods and approaches. This study shows that a MQ approach proves beneficial for increasing the coverage. The PASS2 database, which accounts for SCOP superfamily members that are structurally aligned, becomes a good start point to scan sequence space for good coverage at the complete SCOP superfamily level. Instead of using all SCOP members, using only PASS2 members with a MQ gives rise to good coverage in remote homology detection. In this paper, we have further compared the MQ approach using PSI-BLAST to a MPMQ using PHI-BLAST. The MPMQ approach totally outperforms the MQ approach. Use of patterns makes the initial pattern-hit initiation stringent, thus elevating the specificity of the search ^25^. The study also indicates that a best representative sequence performs better with the PHI-BLAST than the BRS PSI-BLAST while better or equivalent to the MQ PSI-BLAST approach for certain superfamilies. Therefore the use of BRS PHI-BLAST can help save computational time and achieve better coverage. But this is superfamily-specific and for multi-family superfamilies, a query from one family may not be sufficient to associate with hits obtained from a query of a different family within a given superfamily. It is, therefore, necessary to resort to a MQ or MPMQ approach for getting reasonable coverage. The PGC obtained from data mining for all multi-member superfamilies from PASS2 points to the necessity of using multiple queries. The MQ approach is the easiest to follow for an end-user to achieve good coverage at superfamily level, while using MPMQ approach will improve the extent of sequence coverage. However, if there are many members (>10) in a superfamily, MQ approach alone can be adopted. Alternatively, for representative sequences, multiple pattern based PHI-BLAST can be used to achieve a trade-off between computational time and coverage. Thus, different methods and different approaches can be used to improve the sequence searches during remote homology detection.

## References

[1] Thorne JL: Models of protein sequence evolution and their applications. *Curr Opin Genet Dev.* 2000;10(6):602–605. 10.1016/S0959-437X(00)00142-8 11088008

[2] Orengo CA Thornton JM: Protein families and their evolution-a structural perspective. *Annu Rev Biochem.* 2005;74:867–900. 10.1146/annurev.biochem.74.082803.133029 15954844

[3] Lee D Redfern O Orengo C: Predicting protein function from sequence and structure. *Nat Rev Mol Cell Biol.* 2007;8(12):995–1005. 10.1038/nrm2281 18037900

[4] Whisstock JC Lesk AM: Prediction of protein function from protein sequence and structure. *Q Rev Biophys.* 2003;36(3):307–340. 10.1017/S0033583503003901 15029827

[5] Watson JD Laskowski RA Thornton JM: Predicting protein function from sequence and structural data. *Curr Opin Struct Biol.* 2005;15(3):275–284. 10.1016/j.sbi.2005.04.003 15963890

[6] Murzin AG Brenner SE Hubbard T: SCOP: a structural classification of proteins database for the investigation of sequences and structures. *J Mol Biol.* 1995;247(4):536–540. 10.1016/S0022-2836(05)80134-2 7723011

[7] Pugalenthi G: GenDiS: Genomic Distribution of protein structural domain Superfamilies. *Nucleic Acids Res.* 2004;33(Database issue):D252–D255. 10.1093/nar/gki087 15608190 PMC540041

[8] Wistrand M Sonnhammer E: Improved profile HMM performance by assessment of critical algorithmic features in SAM and HMMER. *BMC Bioinformatics.* 2005;6:99. 10.1186/1471-2105-6-99 15831105 PMC1097716

[9] Dessailly BH Redfern OC Cuff A: Exploiting structural classifications for function prediction: towards a domain grammar for protein function. *Curr Opin Struct Biol.* 2009;19(3):349–356. 10.1016/j.sbi.2009.03.009 19398323 PMC2920418

[10] Altschul SF Madden TL Schäffer AA: Gapped BLAST and PSI-BLAST: a new generation of protein database search programs. *Nucleic Acids Res.* 1997;25(17):3389–3402. 10.1093/nar/25.17.3389 9254694 PMC146917

[11] Eddy SR: A new generation of homology search tools based on probabilistic inference. *Genome Inform.* 2009;23(1):205–211. 10.1142/9781848165632_0019 20180275

[12] Johnson LS Eddy SR Portugaly E: Hidden Markov model speed heuristic and iterative HMM search procedure. *BMC Bioinformatics.* 2010;11:431. 10.1186/1471-2105-11-431 20718988 PMC2931519

[13] Zhang Z Miller W Schäffer AA: Protein sequence similarity searches using patterns as seeds. *Nucleic Acids Res.* 1998;26(17):3986–3990. 10.1093/nar/26.17.3986 9705509 PMC147803

[14] Sandhya S Chakrabarti S Abhinandan KR: Assessment of a rigorous transitive profile based search method to detect remotely similar proteins. *J Biomol Struct Dyn.* 2005;23(3):283–298. 10.1080/07391102.2005.10507066 16218755

[15] Rehmsmeier M Vingron M: Phylogenetic information improves homology detection. *Proteins.* 2001;45(4):360–371. 10.1002/prot.1156 11746684

[16] Alam I Dress A Rehmsmeier M: Comparative homology agreement search: an effective combination of homology-search methods. *Proc Natl Acad Sci U S A.* 2004;101(38):13814–13819. 10.1073/pnas.0405612101 15367730 PMC518839

[17] Park J Karplus K Barrett C: Sequence comparisons using multiple sequences detect three times as many remote homologues as pairwise methods. *J Mol Biol.* 1998;284(4):1201–1210. 10.1006/jmbi.1998.2221 9837738

[18] Anand B Gowri VS Srinivasan N: Use of multiple profiles corresponding to a sequence alignment enables effective detection of remote homologues. *Bioinformatics.* 2005;21(12):2821–2826. 10.1093/bioinformatics/bti432 15817691

[19] Bhaduri A Pugalenthi G Sowdhamini R: PASS2: an automated database of protein alignments organised as structural superfamilies. *BMC Bioinformatics.* 2004;5:35. 10.1186/1471-2105-5-35 15059245 PMC407847

[20] Chandonia JM Hon G Walker NS: The ASTRAL Compendium in 2004. *Nucleic Acids Res.* 2004;32(Database issue):189D-192. 10.1093/nar/gkh034 14681391 PMC308768

[21] Larkin MA Blackshields G Brown NP: Clustal W and Clustal X version 2.0. *Bioinformatics.* 2007;23(21):2947–2948. 10.1093/bioinformatics/btm404 17846036

[22] Bairoch A: PROSITE: a dictionary of sites and patterns in proteins. *Nucleic Acids Res.* 1991;19(Suppl)2241–2245. 10.1093/nar/19.suppl.2241 2041810 PMC331358

[23] Mutt E Mitra A Sowdhamini R: Search for Protein Sequence Homologues that Display Considerable Domain Length Variations. *Int J Knowl Dis Bioinform.* 2011;2(2):55–77 10.4018/jkdb.2011040104

[24] Oliveros JC: VENNY. An interactive tool for comparing lists with Venn Diagrams. *BioinfoGP of CNB-CSIC.* 2007 Reference Source

[25] Bhaduri A Ravishankar R Sowdhamini R: Conserved spatially interacting motifs of protein superfamilies: application to fold recognition and function annotation of genome data. *Proteins.* 2004;54(4)657–670. 10.1002/prot.10638 14997562

[26] Lee MM Chan MK Bundschuh R: Simple is beautiful: a straightforward approach to improve the delineation of true and false positives in PSI-BLAST searches. *Bioinformatics.* 2008;24(11):1339–1343. 10.1093/bioinformatics/btn130 18403442

