# Supplementary material for: Improved performance of sequence search approaches in remote homology detection
Source: F1000Res. 2014 Jul 16;2:93. Originally published 2013 Mar 22. [Version 2] doi: 10.12688/f1000research.2-93.v2 (PMC4240247; doi:10.12688/f1000research.2-93.v2)
Supplement: Peer review history

# Peer review: Referee response for version 2

**DOI:** 10.5256/f1000research.4848.r5565

**Published:** 2014-07-28

**Authors:** Saikat Chakrabarti

**Affiliations:** 1 Structural Biology & Bio-Informatics Division, Indian Institute of Chemical Biology, Kolkata, West Bengal, India

The authors have successfully addressed most of the questions raised during the review of version 1. The representation of the data in version 2 explains the outcome of the experiments more satisfactorily.

I have read this submission. I believe that I have an appropriate level of expertise to confirm that it is of an acceptable scientific standard.

# Peer review: Referee response for version 1

**DOI:** 10.5256/f1000research.1249.r3320

**Published:** 2014-04-17

**Authors:** Mallur Srivatsan Madhusudhan

**Affiliations:** 1 Indian Institute of Science Education and Research, Pune (IISER), Pashan, Pune, India

In this study the authors have sought to improve the alignment protocols that make use of standard tools such as PSI-BLAST and PHI-BLAST to find protein sequences that are remotely related to a query sequence. These distant relationships would be useful in annotating protein functions and designing experiments.

The authors of this study use multiple queries (MQ) as a starting point to detect protein homologous relationships. The results from MQ are compared against another search that makes use of multiple patterns deduced from multiple queries (MPMQ). The protocol has been benchmarked on a set of 12 superfamilies consisting of different fold types. The manuscript is clearly presented and the plots and tables make their points clearly and unambiguously.

I wish that the authors would clarify the following points:

1. For any given query sequence what would be the best strategy to adopt: MQ or MPMQ? How would a potential user make this judgement? The prescriptions (conclusions section) from the text are a little ambivalent, especially to a non-expert user/reader. 
2. Demonstrating the efficacy of the search protocol over only 3 examples of each type of fold (alpha, beta, alpha/beta and alpha+beta) may not be sufficient. The authors should look to increase the benchmark size or show that that the current benchmark is statistically representative. While the authors have not discussed this in depth, the implication appears to be that the search results appear to be independent of fold type. Could the authors comment on whether this is true for other types of SCOP fold families (trans-membrane proteins etc)?
3. In the two parts of Figure 2, it is apparent that most PSSMs diverge (non-retention of query), if at all, within the first 50% of the iterations. Only very seldom does this happen afterwards. Is there a lesson one can learn from this result? Could future search strategies make use of this observation? Admittedly not all divergent PSSMs are detrimental to the finding of homologues, but is there a way one can control for this during the search?
4. It would be instructive to have a somewhat more quantitative description of the process in the abstract. I think that reader would benefit in knowing how much better the two protocols are (‘X’ more sequence homologues identified; or TP rate goes up by ‘X’ and FP rate reduces by ‘Y’ - or something along these lines).The same quantifiable measures should also be mentioned in the concluding section.

**Minor points**

1. In the pattern generation sub-section of the Methods section, the authors say that in the event of inadequate number of hits they relax the stringent criteria of allowing hits between 60-90% in sequence identity to include sequences in the range 40-95%. Why is the range 40-95% not used uniformly? Is it the case that the pattern recognition program suffers from a drastic decrease in efficiency when decreasing sequence identity from 60 to 40%?
2. What happens in cases where patterns are >15 amino acids in length? How does the program deal with this? Does it break the patterns into subsets of lengths that have a maximum length of 15?

I have read this submission. I believe that I have an appropriate level of expertise to confirm that it is of an acceptable scientific standard.

# Response

**Published:** 2014-06-11

**Authors:** Ramanathan Sowdhamini

**Affiliations:** National Centre for Biological Sciences, India

We thank Dr. Madhusudhan for providing constructive comments and interests. We had applied these enhanced sequence search strategies to 1961 superfamilies and we find similar trends. We have now mentioned recommended strategies and parameters in the Conclusion section. Having said that, some of the strategies, search directions and parameters depend on the sequence dispersion and population of families and few decisions are highly family-specific.

We have the following responses to specific comments:

**Major query 1:** The MQ approach is easiest to follow as the user needs only the input sequences for a given superfamily. If the superfamily is very diverse with many members (>10), the MQ approach can be adopted, perhaps for representative sequences, to achieve a trade-off between computational time and coverage. We have now mentioned this in Conclusions of the revised manuscript.

**Minor query 1:** It is difficult to obtain patterns qualifying stringent cut-offs followed (please refer to Methods section) when the sequence identity is reduced to 40% that will be applicable for all the superfamilies. It was important to ensure that patterns do reflect conservation, thereby rendering specificity to the PHI-BLAST searches. However, in cases where there were few homologues to deduce a conserved pattern even at 60% sequence identity, 40% sequence identity cut-off had to be employed.

**Minor query 2**:If a pattern is >15 amino acids in length, the long pattern is broken into subsets of patterns which qualify all the stringent cut-offs and have a maximum length of 15.

# Peer review: Referee response for version 1

**DOI:** 10.5256/f1000research.1249.r3732

**Published:** 2014-03-11

**Authors:** Saikat Chakrabarti

**Affiliations:** 1 Structural Biology & Bio-Informatics Division, Indian Institute of Chemical Biology, Kolkata, West Bengal, India

**Concept and strategy of the work:**

In this study the authors have chosen 12 superfamilies from PASS2 database and collected the unique sets of hits generated by PSI-BLAST and PHI-BLAST against the non-redundant protein database (NR-Db) and checked how many of the sequences from the same superfamily are returned. There are quite a number of already published reports that dealt with the same problem but used different dataset and search algorithms. The same research group has already published a report where they have worked improvement of PSI-BLAST using different parameters ^1^^,^^2^. Though neither the concept nor the strategy is novel, the search for a reliable method for detection of remote homologues is crucial. The report provides good information about the performance of PSI-BLAST and PHI-BLAST with a defined set of parameters and also compares their performance. This could be useful information for future researchers who want to use the same homology search algorithm. I believe clarification of the following points will improve the article.

**Major Comments:**

1. The strategies (MQ) and (MPMQ) are not selected by choice. It is an obvious process. If one wants to check the remote homologues in a superfamily level; one has to take all the sequences of the superfamily. Also MP is an obvious requirement for PHI-BLAST. So MPMQ and MQ are rather obvious requirement for running the programs effectively. But it has been reported other way round. 
2. The authors have used a small fraction of the total dataset (12 out of 1961 PASS2 super-families) for demonstration of their algorithm. Testing the algorithm on a larger dataset will be helpful for establishing the efficacy of the algorithm. As mentioned in the manuscript, the result for 1961 families should be produced. Also the basis for choosing only those 12 superfamilies is not clearly mentioned. Would the result be similar for any random 12 superfamilies? Also, will this process return the same robustness if we use the same superfamilies from SCOP, where the number of sequences is greater?
3. The authors have demonstrated the efficacy of the two methods by means of bar graphs (figures 3 and 4), which represent the coverage percentage of each of the approaches of MQ and MPMQ. It would be more informative if the authors provided receiver operating characteristics (ROC) plots to show the rate of detection true positives (TPs) and false positives (FPs) by the methods.
4. In reference to figure 4, the authors should explain a little more about why they have considered the coverage percentage of MPMQ approach as the saturation percentage. In this way the analysis is biased towards PHI-BLAST. In my opinion, the representation of the true coverage in percentage for both methods would be more informative.
5. It is commendable how the authors have been able to identify all possible patterns that might be a property of a superfamily. However, my suggestion would be to use other third party motif-finding programs (apart from MOTIFS for which no published references were provided) and consider the consensus motif predicted by all such software for a particular super-family. Information about those motifs will be helpful. Care should be taken to ensure that the motifs are biologically and statistically relevant.
6. In the Validation section, HMM profiles for all PASS2 superfamilies were made and homologues were scanned against the HMM. The filtering criteria here are not mentioned. They also mention that they have used two-step validations. But actually the second step is used for the hits which fail to associate with any superfamily by level 1 search. So the validation is not two-step but one-step for each set.
7. The title states “ *Improved performance of sequence search algorithms in remote homology detection*” but there is no improvement measure taken for enhancing the performance. And both the algorithms are well established and widely used for remote homology search.  So what is the new improvement of the algorithms made by the authors?
8. On page 6 maximum length of motif is mentioned as 40 and in the Materials and methodology section (page 4) the same is mentioned as 15. Which one has actually been used?
9. In figure 5b, where increase in PGC is compared with number of member in families, the relation between the two is not clear. There are few families with more than 30 members. But only those few families have very high PGC. So a statistical relation is required. From the graph it looks like the algorithm is biased towards multi-member families which contain a high number (more than 30) of members. The efficiency of the program must be tested on single member, two-member and those multimember families which have less than 30 members.

**Minor Comments:-**

1. The authors could throw some light on the fact as to why they have not used all the family members of a SCOP superfamily as has been mentioned in PASS2.  For example, in table 1 the number of families mentioned by the authors for SCOP superfamily 47336 is 7, whereas PASS2 lists at 8.
2. It is not really clear what the coverage of the Best Representative Sequence (BRS) of a superfamily dataset says about the performance of the method.
3. In the collection of the hit pool, unique sets of homologues (hits) for each superfamily were taken. That means each protein in the sets of hits is associated with one query protein of the superfamily protein only. How is that done? The method is not clear.
4. In graph 2A and 2B the y-axes are comparable so they should be in same scale.
5.  Both in figure 3 and 4 if the one-member, two-member and multi-member families were marked separately the result would be more prominent.
6. In the explanation of coverage, where the differences of the superfamilies are explained in terms of their constituent families, actual examples or a case study may be more helpful.

I have read this submission. I believe that I have an appropriate level of expertise to confirm that it is of an acceptable scientific standard, however I have significant reservations, as outlined above.

## References

[1] SandhyaSChakrabartiSAbhinandanKRSowdhaminiRSrinivasanN: Assessment of a rigorous transitive profile based search method to detect remotely similar proteins. *J Biomol Struct Dyn*.2005;23(3) : 93283-29810.1080/07391102.2005.1050706616218755

[2] BhadraRSandhyaSAbhinandanKRChakrabartiSSowdhaminiRSrinivasanN: Cascade PSI-BLAST webserver : a remote homology search tool for relating protein domains. *Nucleic Acids Res*.2006;34(web server issue) : 93143-14610.1093/nar/gkl15716844978PMC1538780

# Response

**Published:** 2014-06-11

**Authors:** Ramanathan Sowdhamini

**Affiliations:** National Centre for Biological Sciences, India

We thank Dr. Saikat Chakrabarti for their useful and encouraging comments. The MQ or MPMQ approach, whilst being obvious choices, are also computationally demanding and this analysis on 12 superfamilies was a conscious attempt to measure the extent of gain/loss of coverage. We have now introduced a Venn diagram that provides a cumulative measure of coverage of these different approaches (Figure 4-A of the revised manuscript). We do find similar trends when applied on the 1961 superfamilies. We had meant this as an approach, not an algorithm, and we have now modified the title as well.

Our responses to specific comments:

**Major Comment 3:** We agree that statistical measures such as ROC curves are often employed to compare methods and approaches. However, since our sequence searches are against the non-redundant sequence database, despite our independent validations, there is no clear negative dataset nor is there is a ‘gold standard’. So it is not possible to measure FPR and compare them for different approaches.

**Major Comment 4**: A comparison of hits by different approaches shows that MPMQ achieves the highest coverage. We now provide a Venn diagram (Figure 4-A of the revised manuscript) that provides a quantitative idea of the performance of different approaches in terms of accumulation of validated hits.

**Major Comment 6**: It is true that the validation is a one-step procedure since single-query HMM validation was invariably performed for validation of all the hits; however, it was necessary only when full-Sf HMM failed to validate.

**Major Comment 7**: We are sorry that the title has been misleading. We had meant to say ‘ *sequence search approaches*’ since there is indeed no new algorithm involved. We have now modified the title. However, the improvement in performance is to arrive at a good balance between sequence coverage and computational time. Our analysis shows that it is possible to obtain good coverage by employing multiple queries followed by multiple patterns for the **best** representative query.

**Minor query 1**:We have used all the members from PASS2 for all the sequence searches. In case of superfamily 47336, the current updated version of PASS2 has 8 members whereas the earlier (2008) version has been used for the analysis of 12 superfamilies. Therefore there is a difference in the number of members.

**Minor query 4**: We are sorry about this and we have now modified Figure 2 in the revised manuscript.

**Minor query 6**: Thanks for this suggestion. We are now including one more example in the revised manuscript to convey practical biological application.
